# Oxygen and SO_2_ Consumption of Different Enological Tannins in Relationship to Their Chemical and Electrochemical Characteristics

**DOI:** 10.1021/acs.jafc.0c00044

**Published:** 2020-03-10

**Authors:** Maurizio Ugliano, Davide Slaghenaufi, Luigi Picariello, Gianmarco Olivieri

**Affiliations:** Department of Biotechnology, University of Verona, Villa Lebrecht, Via della Pieve 70, 37029 San Pietro in Cariano, Italy

**Keywords:** enological tannins, oxidation, SO_2_, voltammetry, ellagitannins, flavan-3-ols

## Abstract

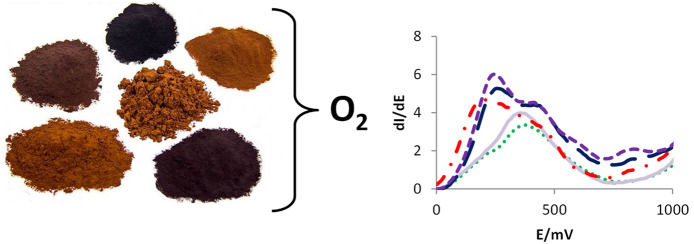

The oxidative behavior of five commercial enological tannins of different sources (tea, grape marc, grape seed, untoasted oak, and toasted oak) was investigated in model wine solutions in the presence or absence of SO_2_. Solutions of the tannins were also analyzed for total phenolics, methyl cellulose precipitable tannins, high-performance liquid chromatography, and linear sweep voltammetry. Tea and oak-derived tannin solutions were characterized by the highest oxygen consumption rates, with oak-derived tannins exhibiting the highest oxygen consumption rates per milligram of phenolic material present. Linear sweep and derivative voltammetry parameters were well-correlated with oxygen consumption rates, whereas total phenolics or total tannins were not. All tannins were associated with consumption of SO_2_ upon reaction with oxygen, with the lowest rate of SO_2_ lost per milligram of O_2_ reacted being observed for oak tannins.

## Introduction

Tannins are naturally occurring compounds of fruits and vegetables that are of primary interest in the food industry for their nutritional, technological, and sensory properties.^1−3^ In the context of winemaking, tannins are considered of major importance to wine quality,^3−5^ being the main drivers of perceived astringency.^6^ Tannins play a central role in wine color and aroma stability during aging.^3,7−10^ Wine tannins are primarily derived from grapes, where they are contained in the skins and in the seeds.^3^ Tannin concentrations in different berry compartments can be affected by a number of different factors, including grape variety,^11^ viticultural conditions, and maturity at harvest (reviewed in refs (3 and 12)). During winemaking, maceration management provides further opportunities to optimize the wine tannin content.^12^ An additional tannin source of enological relevance is provided by different forms of oak commonly used in winemaking, primarily oak barrels and oak chips or staves. Oak is rich in ellagitannins, mostly vescalagin, castalagin, and related derivatives, exhibiting unique chemical and biological activities of enological relevance.^13−15^ As a result of the central role of tannins in wine quality, there is a great emphasis in obtaining wine tannin profiles that are adequate to the different wine styles being produced.^3−6^ However, tannin management at the level of grapes and young and aged wine remains complex as a result of the high number of factors involved, such as variability of tannin evolution patterns during grape maturation, tannin extraction patterns during maceration, and sensory contribution of different tannin fractions.^12^ For this reason, commercial preparations of exogenous tannins are often added in the winery. These are classified as food additives or processing aids having different chemical characteristics (e.g., condensed and hydrolyzable tannins), botanical origin (grape seed or grape skin, oak wood, and exotic wood), and/or preparation process.^16^ Commercial tannins are employed in the winery with a number of different objectives, including clarification/fining, color stabilization, modulation of mouthfeel properties, increase of antioxidant capacity, and inhibition of laccase.^16−19^ The latter aspect, namely, the capacity of tannins to modify the oxidative behavior of wine, is probably among the most important reasons explaining tannin use in the winery, also in consideration of the increasing interest toward the production of wines that can withstand oxidation.^15−20^ However, classification of tannins based on their actual antioxidant capacity remains challenging, also as a result of the fact that different antioxidant assays produce different and sometimes contradictory results.^17,18,21^ At the same time, analysis of the tannin composition by means of advanced analytical chromatographic techniques is also complex and time-consuming.^2,22^ Fingerprinting approaches by means of multiple spectral techniques have also been used, highlighting the difficulty of defining one single approach able to provide a comprehensive classification of tannin multiple chemical and technological properties.^23^ Conversely, other analytical techniques successfully applied to the study of wine phenolics and wine oxidative behavior; for example, electrochemical techniques^24−28^ have received limited interest for tannin analysis. More recently, it was proposed that the actual ability of tannin to consume oxygen is one aspect of the supposed tannin antioxidant capacity that should be further investigated.^17^ At the same time, the ability of condensed tannins to react with SO_2_ during wine aging has also been described,^29^ raising additional interest toward the need to further characterize tannin oxidative response in wine conditions.

The aim of the present study was to investigate oxygen and SO_2_ consumption characteristics of different categories of commercial tannins and to evaluate whether such characteristics could be associated with compositional or electrochemical characteristics of individual tannins.

## Materials and Methods

### Chemicals and Commercial Tannins

Folin–Ciocalteu reagent, sodium carbonate, methyl cellulose, ammonium sulfate, gallic acid, and catechin were obtained from Sigma. Epicatechin, procyanidin B2, and epigallocatechin gallate were obtained from Extrasynthese (Lyon, France). Five different commercial enological tannins were studied. All tannins were provided by Enologica Vason (Pescantina, Verona, Italy) and were obtained from one of the following matrices: green tea (later labeled as tea), grape marc (including skins, seeds, and pulp solids), grape seed, not toasted French oak (later labeled as oak not toasted), and toasted French oak (later labeled as oak toasted). Tannins were dissolved at 500 mg/L in model wine solutions containing 12% ethanol, 5 g/L tartaric acid, 5 mg/L iron (added as FeSO_4_·7H_2_O), and 0.5 mg/L copper (added as CuSO_4_·5H_2_O), with pH adjusted to 3.2 by means of NaOH. SO_2_ was added as potassium metabisulfite where required, to a final concentration of 30 mg/L free SO_2_. Metals were added to catalyze the oxidative reaction of *ortho*-dipehnol compounds,^17^ considering the central role of this mechanism in wine oxidation.^20^

### Oxidation Experiments

Tannin solutions were air-saturated and placed in 115 mL clear glass vials fitted with Pst3 oxygen sensors (Nomacorc, Thimister, Belgium), crimped without leaving any headspace, and sealed with Araldite glue. After 40 min from filling, dissolved oxygen was measured by means of a Nomasense P300 oxygen analyzer (Nomacorc, Thimister, Belgium) to obtain the initial oxygen content of the samples. Sample vials were then placed at 25 °C, and dissolved oxygen content was monitored daily. A series of analogue samples, which were not air-saturated and had an initial dissolved oxygen content lower than 200 μg/L, was also prepared. Upon consumption of 5 ± 0.1 mg/L oxygen, samples of oxygenated solutions opened and analyzed, so that all solutions had consumed an equal amount of oxygen within a reasonably short and similar time frame. At this same time, samples of the corresponding non-oxygenated controls were also opened and submitted to analyses. All experiments were carried out in duplicate. Oxygen consumption rates (OCRs) were obtained by dividing the amount of oxygen consumed in a given time frame by the length of the time frame. Accordingly, initial OCR was calculated at 24 h, along with average OCR when consumption of 5 ± 0.1 mg/L oxygen was recorded.

### Chemical and Electrochemical Analyses

Voltammetric analyses were performed with a Palmsens potentiostat (Palmsens, Netherlands) using disposable screen-printed sensors in a three-electrode arrangement (Nomacorc, Thimister, Belgium). The working electrode (WE) was a screen-printed carbon paste electrode operating in conjunction with a screen-printed carbon paste counter electrode and a silver/silver chloride (Ag/AgCl) reference electrode. The analytical procedure has been described elsewhere.^28^ Briefly, a drop of sample at 22 °C with no preliminary sample dilution was loaded onto a sensor, and linear sweep voltammograms were acquired between 0 and 1000 mV at a scan rate of 100 mV/s. After each measurement, the sensor was discarded and a new sensor was used. All measurements were carried out in duplicate. All potentials are reported against the Ag/AgCl reference electrode. Derivative voltammograms were obtained with The Unscrambler (Camo, Norway), applying a 10 point Savitzky–Golay smoothing.

Free and total SO_2_ measurements were carried out using a Biosystems multiparametric analyzer and the dedicated kit (Biosystems, Spain). Th limit of detection of the method used was 3 mg/L, while the limit of quantification was 5 mg/L.

Total phenolic index (TPI) and methyl cellulose precipitable tannins (MCPTs) were determined as previously described,^4,18^ with MCPT analysis being carried out directly on tannin solutions by means of the addition of a methyl cellulose solution and saturated ammonium sulfate.

High-performance liquid chromatography (HPLC) separation and quantification of phenolic compounds was carried out according to ref (30). Analyses were performed using a HPLC Jasco LC-2000 Plus (JASCO, Inc., Easton, MD, U.S.A.), consisting of a LC-Net II/ADC system controller, AS-2055 autosampler, PU-2085 quaternary gradient pumps, CO-2060 column ovens, and MD-2010 diode array. Samples (20 μL) were loaded onto a Agilent PLRP-S 100 Å reversed-phase polystyrene divinylbenzene column (4.6 × 150 mm, 3 μm particle size) protected with a guard cartridge with the same packing material (PLRP-S, 5 × 3 mm) kept at 35 ± 1 °C used as the stationary phase. The HPLC solvents were solvent A consisting of 1.5% (v/v) *ortho*-phosphoric acid (EMP Chemicals, Gibbstown, NJ, U.S.A.) and solvent B consisting of 80% acetonitrile (HPLC grade, Honeywell, Muskegon, MI, U.S.A.) with 20% solvent A. The following gradient was established: 0 min, 6% B; 73 min, 31% B; 78 min, 62% B, staying constant until 86 min; and 90 min, 6% B. This zero-time solvent mixture was followed by a 15 min equilibrium period prior to injecting the next sample. The flow rate of the mobile phase was 1 mL/min. A total of 20 μL of calibration standards was injected onto the column. All of the samples were filtered through 0.20 μm Microliter polytetrafluoroethylene (PTFE) membrane filters (Wheaton, NJ, U.S.A.) into dark glass vials and immediately injected into the HPLC system. Detection was carried out by monitoring the absorbance signals at 280 nm and identified by comparison to retention times of standards.

### Statistical Analysis

Analysis of variance was carried out on all data, and means were compared by Tukey’s test. Analyses were performed using XLSTAT (version 2013.6.04, Addinsoft, Paris, France).

## Results

Compositional parameters of the five different enological tannins studied are shown in Table 1. Tea tannins were richer in total phenolics as well as MCPTs (expressed as grams per 100 g of commercial product). Grape marc and grape seed tannins showed intermediate values, whereas lower values were observed for both oak tannins. In comparison to oak tannins, tea and both grape-derived tannins were characterized by a higher content of catechin, epicatechin, gallic acid, epigallocatechin gallate, and procyanidin B2, the dimer of epicatechin. Semi-quantitative analysis of the broad peak corresponding to unresolved polymeric compounds^22^ indicated that oak-derived and grape seed tannins were much richer in this fraction, whereas tea tannins were the least rich.

**Table 1:** Chemical Composition of the Analyzed Tannins

	TPI (%)^a^	MCPT (%)^a^	catechin (mg/L)	epicatechin (mg/L)	gallic acid (m/L)	epigallocatechin gallate (mg/L)	procyanidin B2 (mg/L)	polimeric material (mg/L)^b^
tea	88.1 ± 1.8 d	87.8 ± 3.4 e	73.2 ± 1.4 d	42.7 ± 1.3 d	19.9 ± 1.2 d	133.2 ± 0.8 c	286.3 ± 6.9 d	47.4 ± 3.4 a
grape marc	52.7 ± 1.0 b	37.5 ± 0.8 c	23.0 ± 0.7 b	11.5 ± 1.2 b	6.5 ± 0.4 b	0.7 ± 0.1 b	2.4 ± 0.4 b	122.1 ± 5.8 b
grape seeds	58.3 ± 0.4 c	45.6 ± 2.1 d	38.2 ± 1.2 c	21.4 ± 0.8 c	9.2 ± 0.2 c	0.6 ± 0.1 b	5.3 ± 0.4 c	195.0 ± 4.6 c
oak (not toasted)	51.0 ± 0.8 b	32.0 ± 1.1b	1.4 ± 0.3 a	2.1 ± 0.5 a	0.4 ± 0.2 a	0.9 ± 0.0 a	4.1 ± 0.2 a	196.8 ± 1.7 c
oak (toasted)	47.7 ± 1.1 a	24.9 ± 0.9 a	1.6 ± 0.3 a	1.3 ± 0.3 a	0.6 ± 0.1 a	0.9 ± 0.0 a	3.9 ± 0.2 a	221.3 ± 10.3 d

a Values indicate percentage richness (grams per 100 g of product) expressed as TPI or MCPT.

b Quantified as milligrams per liter equivalents of procyanidin B2. With each analytical parameter, different letters denote statistically significant difference at *p* < 0.05.

**Figure 1:**
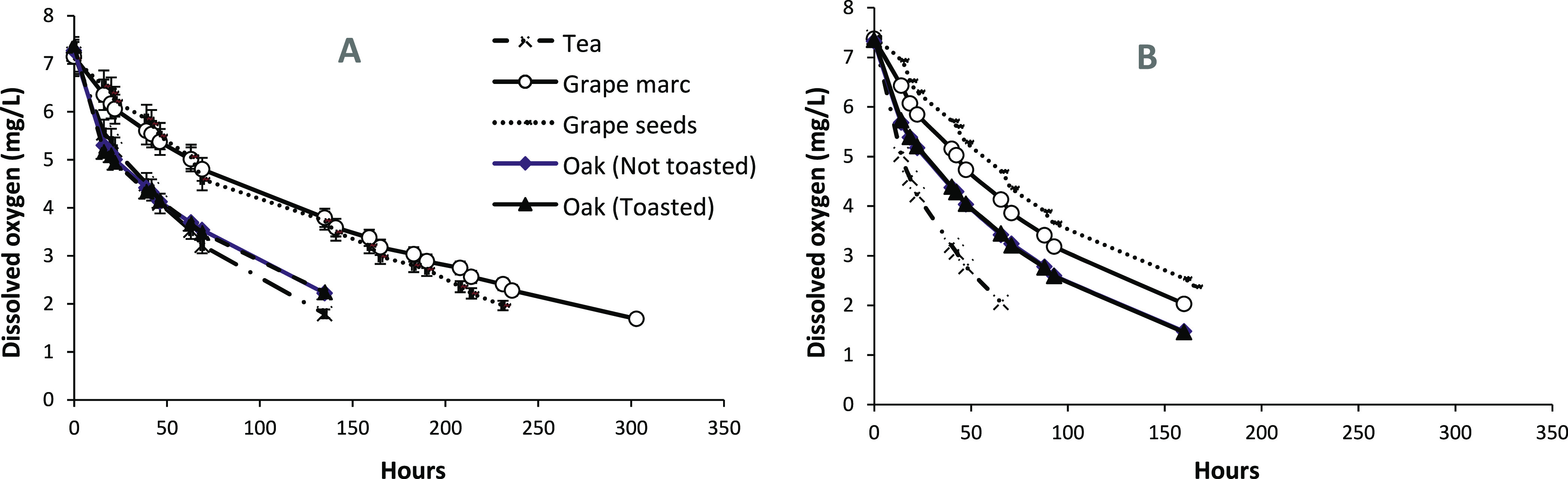
Evolution of dissolved oxygen during oxidation experiments (A) with or (B) without SO_2_.

**Figure 2:**
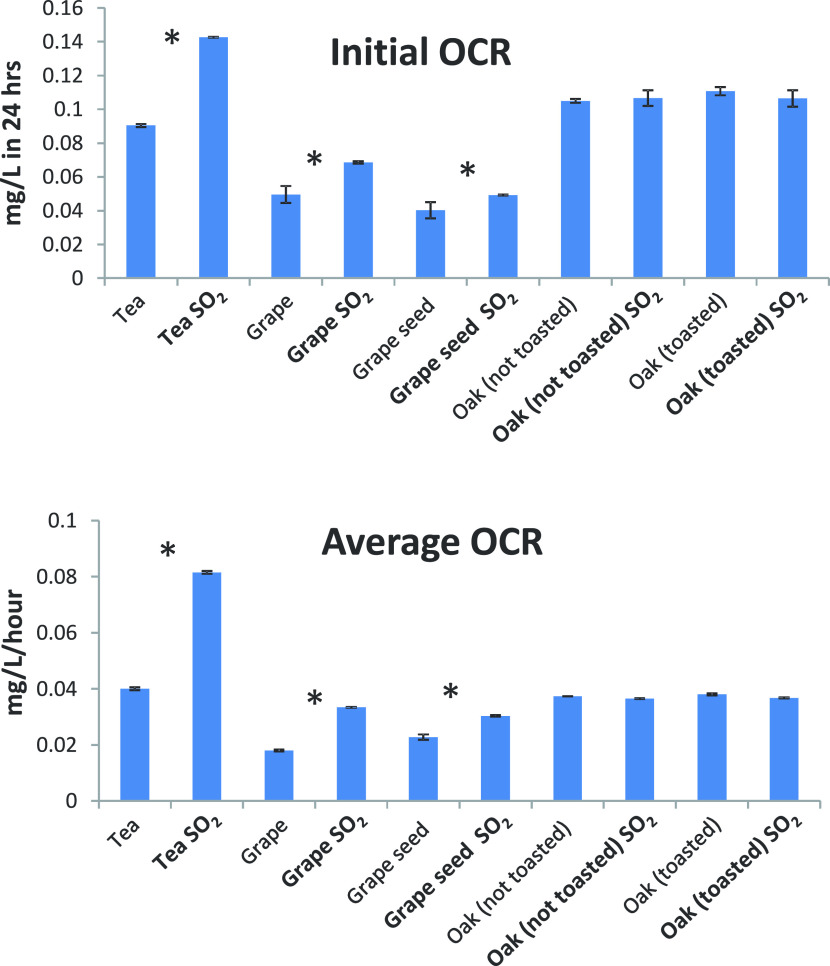
Initial and average OCRs of different tannins in the presence or absence of SO_2_. Within each pair of values asterisks denote statistically significant difference as a result of SO_2_ at *p* < 0.05.

Profiles of oxygen consumption in the presence or absence of SO_2_ are shown in Figure 1, while the relevant kinetic parameters, namely, initial and average OCRs are displayed in Figure 2. The initial OCR indicates the rate of oxygen consumption during the first 24 h, while the average OCR refers to the rate of oxygen consumption for the entire duration of the experiment. At a general level, oak and tea tannins exhibited significantly higher OCRs compared to grape marc and grape seed tannins. The addition of SO_2_ to the model wine solution induced a generalized increase in OCRs for tea, grape marc, and grape seed tannins, which was particularly significant for tea tannins, showing an increase in average OCR of approximately 100%. Conversely, in the case of oak-derived tannins, SO_2_ did not impact OCRs. In no case, an influence of toasting on OCRs was observed for oak tannins.

**Figure 3:**
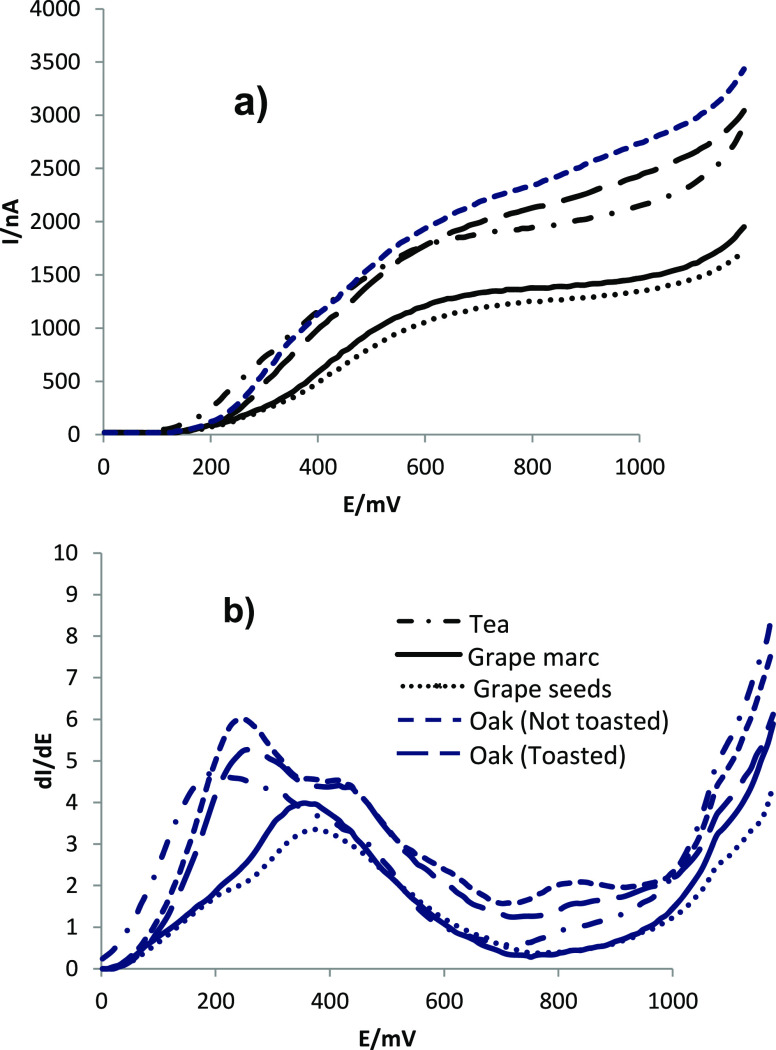
(a) Linear sweep and (b) first derivative voltammograms of tannins.

Linear sweep and first derivative voltammograms of the different products are shown in Figure 3. Raw voltammograms were generally characterized by a large unresolved anodic wave, with tea tannins exhibiting the anticipated onset of anodic oxidation compared to other tannins. Anodic current values were generally higher for tea tannins on the 0–400 mV range, whereas both oak tannins exhibited greater current values above 600 mV. Lower current values were generally observed across the entire potential range for grape marc and grape seed tannins compared to other products. First derivative voltammograms were generally richer than raw voltammograms, with the presence of various features, in particular, one major peak in the 190–250 mV range, which was mostly characteristic of tea tannins, and a second wave in the 374–420 mV region, which was primarily associated with grape marc and grape seed tannins, although it could also be clearly observed oak tannins.

## Discussion

The purpose of this study was to investigate the relationship between compositional characteristics of different sources of commercial tannins and their behavior in oxidative conditions. Two different reaction environments were created by means of adding SO_2_ or not to the model wine solution, so that interactions between tannins and SO_2_ during oxidation could also be studied. With SO_2_ being the most widely used wine antioxidant, this allowed also to evaluate tannin antioxidant capacity in wine-like oxidative conditions. In agreement with previous findings,^17,18,21^ commercial tannins varied significantly in terms of major compositional parameters, reflecting the characteristics of the matrix from which they are derived. Tea, for example, is very rich in flavanols and galloylated derivatives,^31,32^ whereas tannins of different grape sources are a mix of flavan-3-ols, such as catechin and epicatechin, as well as oligomers and larger polymerized forms with subunits of (+)-catechin (C), (−)-epicatechin (EC), (−)-epigallocatechin (EGC), or (−)-epicatechin gallate.^2,3,7^ Conversely, oak is known to be rich in ellagitannins, such as vescalalgin and castalagin.^13,14^ Variations in phenolic and tannin richness of the different commercial enological tannins studied here are in agreement with previous observations, indicating that oak tannins are generally characterized by lower richness values for both of these indicators.^17,18^ HPLC data are also in line with those reported by others, showing an increased content of flavanols in grape-derived tannins compared to oak tannins.^18,33^ Enological tannins derived from tea have not been characterized extensively, although our results are in line with previous reports concerning tea composition.^31^

Despite the well-established relevance of electrochemical methods for the study of a phenolic antioxidant in wine and other matrices,^24−28,32^ applications of electrochemical techniques to the study of commercial tannins are very limited and restricted to cyclic voltammetry.^34^ In the present study, a simple electrochemical approach has been adopted on the basis of linear sweep voltammetry combined with the use of disposable carbon paste sensors,^28^ allowing for a rapid acquisition of voltammograms representative of the anodic oxidation of the different components present in the tannin solutions (Figure 3a). These raw voltammograms allowed for the differentiation of the products into three main groups, essentially based on the potential onset of anodic oxidation and anodic current values and total passed current. Accordingly, tea tannins were characterized by lower potential of the oxidation onset and high anodic current values. Oak-derived tannins exhibited equally high current values, but the onset of oxidation occurred later, whereas grape-derived tannins showed potential oxidation onset similar to oak tannins but a further decrease in anodic current values across the entire potential range. However, raw voltammograms showed, in large part, unresolved signals, so that it was difficult to further explore existing differences and establish relationships between voltammetric and compositional features. Conversely, the derivative voltammogram (Figure 3b) provided useful insights on the electrochemical signature of different tannins, in agreement with previous reports, indicating the potential of this technique for wine analysis.^35^ In particular, the peak at 190 mV observed for tea tannins can be ascribed to richness in highly oxidizable substrates, such as epigallocatechin gallate, which has been shown to oxidize at the surface of carbon electrodes earlier than other readily oxidizable compounds, including catechin and epicatechin.^27,32^ High contents in other highly oxidizable compounds, such as catechin and epicatechin (Table 1), oxidizing at the surface of the carbon paste sensors in the potential range immediately following epigallocatechin gallate oxidation, would explain the unresolved broad peak observed in first derivative voltammograms of tea tannins. Although much less prominent, an early oxidation peak (around 190 mV) was also observed in grape marc and grape seed tannins. Derivative voltammograms of grape-derived tannins were however characterized primarily by a major peak in the 350–360 mV region, attributable to gallic acid as well as other *ortho*-diphenol compounds potentally present in grape marc extracts, such as protocatechuic acid.^36^ In the case of oak tannins, both toasted and not toasted products were characterized by a major peak in the 230–250 mV region, with a marked shoulder around 420 mV. The first peak could be attributed to gallate and ellagic acid moieties of ellagitannins,^34,37^ although more detailed further investigations on ellagitannin electrochemical behavior would be necessary.

**Figure 4:**
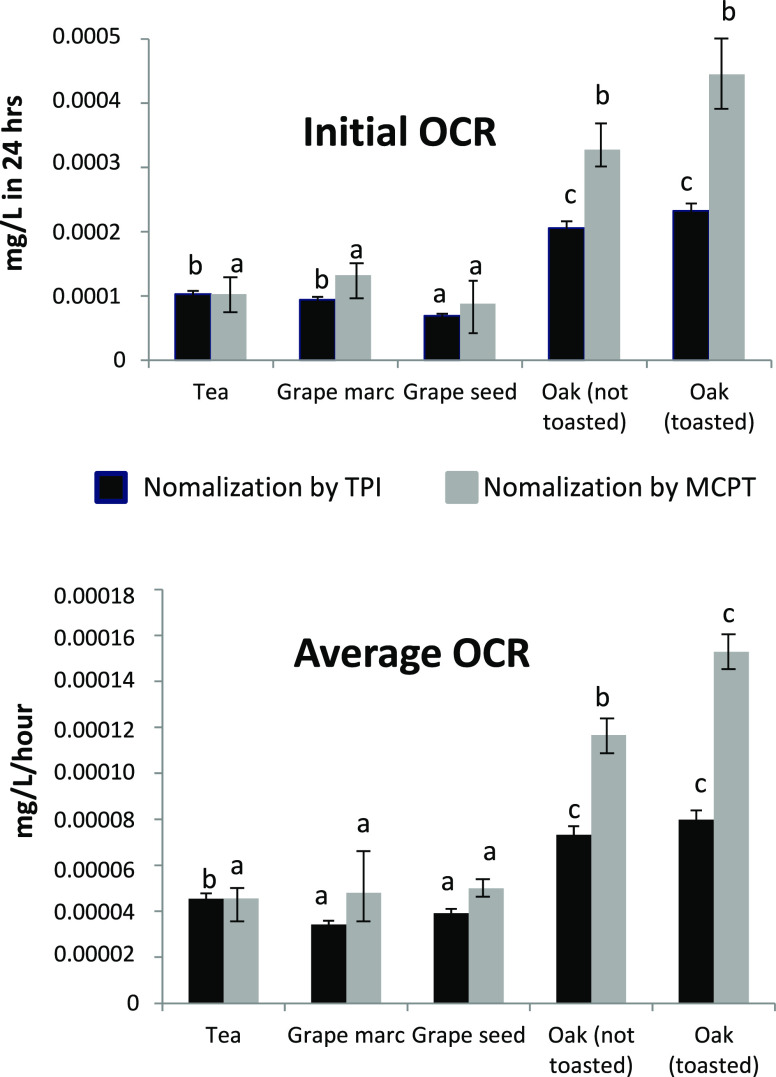
Initial and average OCR values of different tannins after normalization by either TPI or MCPT. Within each series of data (TPI or MCPT normalization), different letters denote statistically significant difference at *p* < 0.05.

The data concerning OCRs indicated that the compositional differences across the range of tannins studied can significantly affect the ability of the different tannins to react with oxygen. In this respect, our results confirm the greater oxygen reactivity of oak tannins compared to grape-derived tannins,^17,18^ also highlighting the high oxygen reactivity of tea tannins in wine-like conditions, which was not previously reported to our knowledge. Although OCR values indicated similar oxygen reactivity for tea and oak tannins, the fact that these products differ substantially for total phenolic and total tannin richness (Table 1) deserves further attention, because tannin OCRs are strongly affected by the concentration of oxidizable substrates.^17^ Accordingly, OCR data obtained in the absence of SO_2_ were normalized by both TPI and MCPT values, and the results are shown in Figure 4. Once normalized by the actual content of phenolic or tannic compounds, it appeared clear that the ability of oak tannins to consume oxygen was much greater, with values up to 4 times higher than those of tea tannins. With tea tannins containing higher concentrations of readily oxidizable substrates, such as catechin, epicatechin, and their galloylated derivatives, it can be assumed that their ability to consume oxygen is primarily associated with oxidation of these *ortho*-diphenol compounds to the corresponding quinones, which will then react with the other phenolic compounds present.^9,10,20^ Conversely, in oak tannins, oxygen-reactive *ortho*-diphenols are associated with the complex structures of vescalagin and castalagin and the related flavano derivatives, with all of them being engaged in inter- and intramolecular oxidoreductions, in which the pyrogallol unit is reversibly converted to semiquinone and quinones.^13,38−40^ In consideration of the recently highlighted importance of understanding the drivers of OCRs,^41,42^ the relationship between OCRs and tannin TPI, MCPT, and electrochemical features was further investigated by assessing the correlation between different pairs of parameters. Each correlation was build using a 10 point data set, and *p* values (Pearson) were calculated with a significance threshold of 0.05. Good correlations were observed between OCRs and electrochemical parameters, such as the total passed current, measured as the area under the curve of linear sweep voltammograms, with *r*^2^ values greater than 0.8 and a high level of significance (Table 2). The potential of the main peak of the first derivative voltammograms was also well-correlated with ORCs. Conversely, correlation coefficients were extremely low for TPI and MCPT, indicating that these parameters were not representative of the ability of tannin to consume oxygen. These observations are in agreement with the data of Gonzalez et al.^35^ for oxidation of white wines. Likewise, as reported by the same authors, linear sweep voltammetry combined with derivative signal treatment can provide information concerning OCRs of complex antioxidant matrices, probably as a result of the ability of this analytical technique to describe the behavior of different oxidizable substrates during oxidation.^43^

**Table 2:** Correlation Parameters for OCRs and Chemical and Electrochemical Parameters

	initial OCR		average OCR	
	*r*^2^	*p*	*r*^2^	*p*
TPI (mg/L)	0.0001	0.926	0.0032	0.142
MCPT (mg/L)	0.0001	0.514	0.0121	0.553
AUC (μc)^a^	0.8402	**<0.0001**	0.8112	**0.000**
main peak (mV)^b^	0.7309	**0.006**	0.8377	**<0.0001**

a Area under the curve of raw voltammograms in 0–1000 mV.

b First derivative voltammogram. Values in bold indicate statistical significance at 0.05.

**Figure 5:**
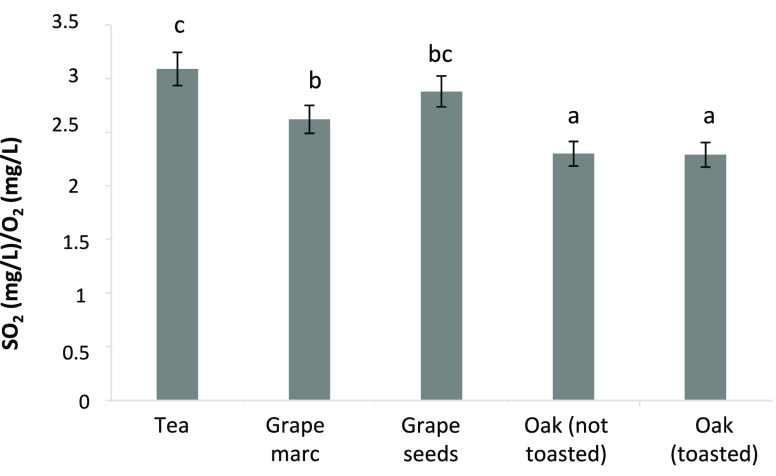
SO_2_/O_2_ ratios of the studied tannins during oxidation. Different letters denote statistically significant difference at *p* < 0.05.

SO_2_ is a strong nucleophile^9^ commonly employed in winemaking for its antioxidant and antimicrobial effects. Under oxidative conditions, SO_2_ is consumed to a large extent,^44,45^ with the consequent decrease of its protective action. In view of the complex relationship between wine composition and SO_2_ oxidative loss,^44,45^ there is great interest in understanding the chemical factors modulating SO_2_ consumption in wine. In the case of the studied tannins, SO_2_ had a major impact on OCRs of tea and grape-derived products, whereas no effect was observed for oak tannins (Figure 2). The ability of SO_2_ to increase OCR has been previously reported^44^ and can be ascribed to the fact that SO_2_ is able to remove the intermediates arising from initial oxidation of the most readily oxidizable compounds, promoting further progress of oxidation reactions. In particular, SO_2_ can reduce oxidation-derived quinones to their original *ortho*-diphenol forms^44^ as well as forming sulfonates of flavan-3-ols or tannins.^28,46,47^ Additional consumption of SO_2_ can arise from reduction to water of hydrogen peroxide arising from ethanol oxidation. Of particular interest in our case was the observation that the SO_2_ influence varied significantly according to the type of tannin, with oak tannin OCRs (both initial and average) not being affected by SO_2_. On the basis of the above-described reaction mechanisms, the strong impact of SO_2_ on OCRs of grape and tea tannins appears somewhat logical because these products were rich in flavan-3-ols, gallic acid, and related derivatives, which are all strongly involved in SO_2_ oxidative loss. As for the apparent lack of influence of SO_2_ on oak tannin OCRs, it can be supposed that, with OCRs of these products already being relatively high, the SO_2_ contribution was marginal. The fast intra- and intermolecular abilities of ellagitannins to reduce oxidation-derived quinones could have therefore limited the involvement of SO_2_ in the oxidative reaction cascade. Further insights in the specificity of SO_2_ behaviors in the presence of different tannins were gained by calculating SO_2_/O_2_ ratios, namely, the amount of SO_2_ lost per milligram of O_2_ reacted. Under ideal reaction conditions, oxidation of an *ortho*-diphenol involves consumption of two SO_2_ for each oxygen reacted, resulting in a mass ratio of 4:1.^45,47^ However, in complex matrices, such as wine or even commercial tannins, the presence of different nucleophiles can trigger competing quinone-consuming reactions,^9^ resulting in deviations from this ideal behavior. Figure 5 shows the SO_2_/O_2_ ratios of the different tannins. Values around 3 were observed for tea and grape seed tannins, progressively decreasing to 2.6 and 2.3 for grape marc tannins and oak tannins, respectively. A highly positive correlation between SO_2_/O_2_ ratios and the sum of flavanols (catechin + epicatechin) and gallic acid was observed (*r*^2^ = 0.92). Generally speaking, these results indicate that, at least in a model wine system, all tested tannins were able to induce a significant SO_2_ loss, which winemakers should bear in mind in consideration of the fact that commercial tannins are often added as antioxidants.^17,18^ In addition, the differences in SO_2_/O_2_ ratios indicate that, within this generalized capacity to induce SO_2_ consumption upon reaction with oxygen, certain tannins, in particular, those containing a high proportion of readily oxidizable flavan-3-ols, are more likely to consume SO_2_, whereas ellagitannins are less prone to induce SO_2_ loss.

In conclusion, this study allowed for the elucidation of certain key aspects of the relationship between the composition of commercial tannins and their ability to consume oxygen and degrade/preserve SO_2_. The data obtained indicated that certain tannins, in particular, those derived from oak, have the ability to rapidly consume oxygen with a relatively reduced decline in the SO_2_ content. This important characteristic should be further investigated in consideration of the ongoing interest toward strategies to reduce SO_2_ demand. Also, the fact that toasting did seem to have a negligible role on this deserves further attention, in view of the contrasting results reported elsewhere.^48^ Tea tannins are also capable of rapidly consuming oxygen, although this is associated with the increased decline in the SO_2_ content, with the latter also being characteristic of grape seed tannins. A high content of flavan-3-ol and gallic acid appeared to be associated with increased SO_2_ consumption per milligram of oxygen reacted. The possibility of classifying the oxygen-consuming capacity of different tannins by a rapid and user-friendly electrochemical approach is reported here for the first time, opening new opportunities for improved control in commercial tannin production and use.
